# Formative evaluation of an existing community-based intervention for alcohol addiction in the Soliga tribal community in Chamarajanagar, Karnataka

**DOI:** 10.12688/wellcomeopenres.24075.1

**Published:** 2026-01-07

**Authors:** Alen Chandy Alexander, Rahul Amruthapuri, Teddy Andrews Jaihind Jothikaran, Prashanth N. Srinivas

**Affiliations:** 1 Center for Training, Research & Innovation in Tribal Health, Institute of Public Health, Bengaluru, Karnataka, India; 2 Department of Social & Health Innovation, Prasanna School of Public Health, Manipal Academy of Higher Education, Manipal, Karnataka, India

**Keywords:** Alcohol addiction recovery, community-based intervention, Soliga tribe, mixed-methods study, culturally sensitive care, peer-led support, formative evaluation, implementation out-comes, short-term patient outcomes.

## Abstract

**Background:**

Socioeconomic stressors, cultural transitions, and limited access to healthcare often influence alcohol addiction among Indigenous communities. The Soliga tribal community lacks culturally tailored de-addiction services in the Chamarajanagar district of Karnataka, India. The Hosa Jeevana (HJ) program is a community-based intervention that integrates clinical care, peer-led support, and culturally relevant awareness strategies to support addiction recovery. This study protocol outlines the plan for evaluating the HJ program’s acceptability, adoption, and short-term patient outcomes through a formative approach.

**Study setting:**

The study will be conducted in the HJ clinic and 17 Soliga settlements in B.R. Hills, Karnataka, India, where the intervention is implemented.

**Method:**

This is an embedded mixed-methods study utilising both quantitative and qualitative approaches. In the first phase, clinical records, Alcohol Use Disorders Identification Test (AUDIT) scores, and program retention rates will be analysed to assess engagement, participation, and early indicators of recovery. In the second phase, in-depth interviews will explore facilitators and barriers to care-seeking and perceptions of program acceptability and adoption among Soliga individuals enrolled in the HJ program, HJ Champions (peer mentors), and other stakeholders such as community health workers and physicians. The qualitative findings will contextualise the trends observed in the quantitative analysis.

**Expected outcomes:**

Implementation outcomes, i.e. acceptability, adoption and identification of barriers and facilitators of the program, are the expected outcomes of the study protocol.

**Discussion:**

The study findings will help refine the existing community-driven addiction recovery program and inform scaling up the intervention. While long-term sustainability falls outside the study’s scope, insights from this evaluation will lay the foundation for future research and policy integration.

## Plain language summary

Alcohol addiction is a significant health concern in many communities, including among Adivasi populations in southern Karnataka. However, existing healthcare services often struggle to reach and support individuals dealing with alcohol dependence effectively. This study aims to introduce and evaluate a culturally responsive intervention program called Hosa Jeevana (meaning "New Life"), explicitly designed for the Solega Adivasi people. The program offers support through health center-based services and community-based efforts. It includes mobile phone-based peer support groups, guidance from abstinent champions, awareness materials in local languages, and access to a 12-hour helpline staffed by an Adivasi counsellor.

The study will assess whether the program is acceptable and valuable to the community by tracking how well it is adopted and whether it helps people reduce or stop harmful alcohol use. By working alongside community members, the research aims to promote accessible, sustainable, and culturally appropriate care pathways, potentially shaping future health policies and services for Adivasi communities.

## Introduction

Research on alcohol use disorders (AUDs) among indigenous and tribal communities highlights structural and social determinants of health that exacerbate addiction. Researchers have found that socioeconomic stressors, cultural displacement, and limited healthcare access often contribute to alcohol use ^1, 2^. The incidence of tobacco and alcohol use in various tribal communities across India is twice as high as that in nearby non-tribal communities ^3^, indicating the need for treatment options.

However, current de-addiction efforts in India primarily focus on urban or facility-based models, often failing to reach marginalised populations. More specifically, de-addiction and cessation services are yet to reach rural areas ^4^. In addition, social determinants of health, like livelihood stress and social exclusion, negatively impact these communities, resulting in poorer treatment outcomes. The lack of context-specific public health programmes further compounds this issue ^5, 6^. Research has demonstrated that generic addiction care models often fail to address the specific barriers faced by tribal populations, such as stigma, mistrust of formal healthcare systems, and a lack of culturally relevant materials and approaches ^7, 8^.

According to global evidence, community-based addiction programs incorporating local cultural elements and peer support structures have demonstrated better outcomes than conventional clinical interventions ^9^. Culturally sensitive care is healthcare that is respectful of and responsive to the cultural beliefs, practices, and needs of diverse patient populations ^10^. It involves tailoring interventions to align with the target community’s social, linguistic, and cultural contexts. This approach improves health outcomes by fostering trust, enhancing patient-provider communication, and increasing the acceptability of healthcare services ^11^.

International models, such as Indigenous-led recovery programs in Canada and Australia, emphasise the role of peer champions and culturally appropriate interventions in addiction care ^12, 13^. Such approaches enhance the adoption and sustainability of interventions in tribal and marginalised populations ^14^. The World Health Organisation (WHO) supports such models, noting that interventions that leverage community participation and local knowledge tend to yield higher engagement rates and long-term adherence to treatment ^15^.

The sociocultural contexts of tribal communities are not being well-characterised enough to design community-based interventions ^16^. As a result, a significant research gap remains, and very limited empirical evidence exists on community-based, culturally sensitive de-addiction interventions in India, especially among Adivasi populations. In this paper, we describe how a team of healthcare staff and workers based in a tribal hospital and community settlements are implementing a clinic and community-based intervention package in B.R. Hills, Chamarajanagar district and present the plan for a formative evaluation of the intervention.

In this manuscript, we use the term *"tribal" and “Adivasi”* to refer to communities officially recognised as *Scheduled Tribes* under the Constitution of India. While we acknowledge that *"Indigenous"* is a preferred term in international discourse, *"tribal"* remains the term commonly used in Indian policy, legal, and programmatic contexts, including in health policy documents, census classification, and welfare schemes and *“Adivasi”* is the term commonly used by such populations in the South Indian context. This term reflects the legal identity and administrative categorisation under which these communities access state benefits and protections. We intend to use the term respectfully and contextually, aligning with how community members and state institutions describe and engage with these populations.

### History of Hosa Jeevana

Based on experiences with and the requests from the Soliga community to address substance use among the Soliga community, one of the authors (PNS) started the HJ clinic, a clinic for mental health and deaddiction services, in 2021. Initially, PNS provided services to a community health worker at the Vivekananda Tribal Health Center for individuals from the tribal settlements in the B. R. Hills region. PNS and the community health worker ran it as a half-day clinic, screening individuals coming to the clinic for mental health issues and substance use disorders and providing clinical care for those who needed it.

Subsequently, with the idea of improving the accessibility for the Soliga community members from other taluks and aligning it with the existing healthcare systems for deaddiction services, the clinic signed an MoU with a nearby taluk hospital and shifted to their campus. However, the team observed that the Taluk hospital staff, primarily from a non-tribal background, did not take ownership of the clinic activities. The clinic appeared distant from the hospital’s existing services. The experiences of the Adivasi patients while approaching the hospital for deaddiction services reflected this distance.

As a result, the clinic returned to its earlier premises at the tribal hospital as a weekly clinic for clinical care, with the community health worker to follow up with the patients and clinician-led patient group meetings. There was a lack of consistency with patient attendance for the meetings. The team questioned whether the lack of attendance was due to a lack of interest in engaging, since the moderator was a clinician and not someone with a lived experience from the community with whom they could relate and share openly.

Reflecting on this, the team decided to reimagine the intervention as a community-driven intervention that seeks to provide a holistic model of alcohol addiction recovery.

### Redesigned Hosa Jeevana

The redesigned intervention contains clinical and community-level activities ( Figure 1). In redesigning HJ, culturally sensitive care was considered through discussions with stakeholders from the Soliga community, incorporating their inputs, traditions, and preferences. Some aspects of the intervention that are culturally sensitive include:

1. Peer-led support groups facilitated by community champions familiar with Soliga traditions.
2. Culturally relevant educational materials, including visual and oral formats in the local language.
3. A focus on participatory methods to ensure that interventions resonate with community values.

**Figure 1.**
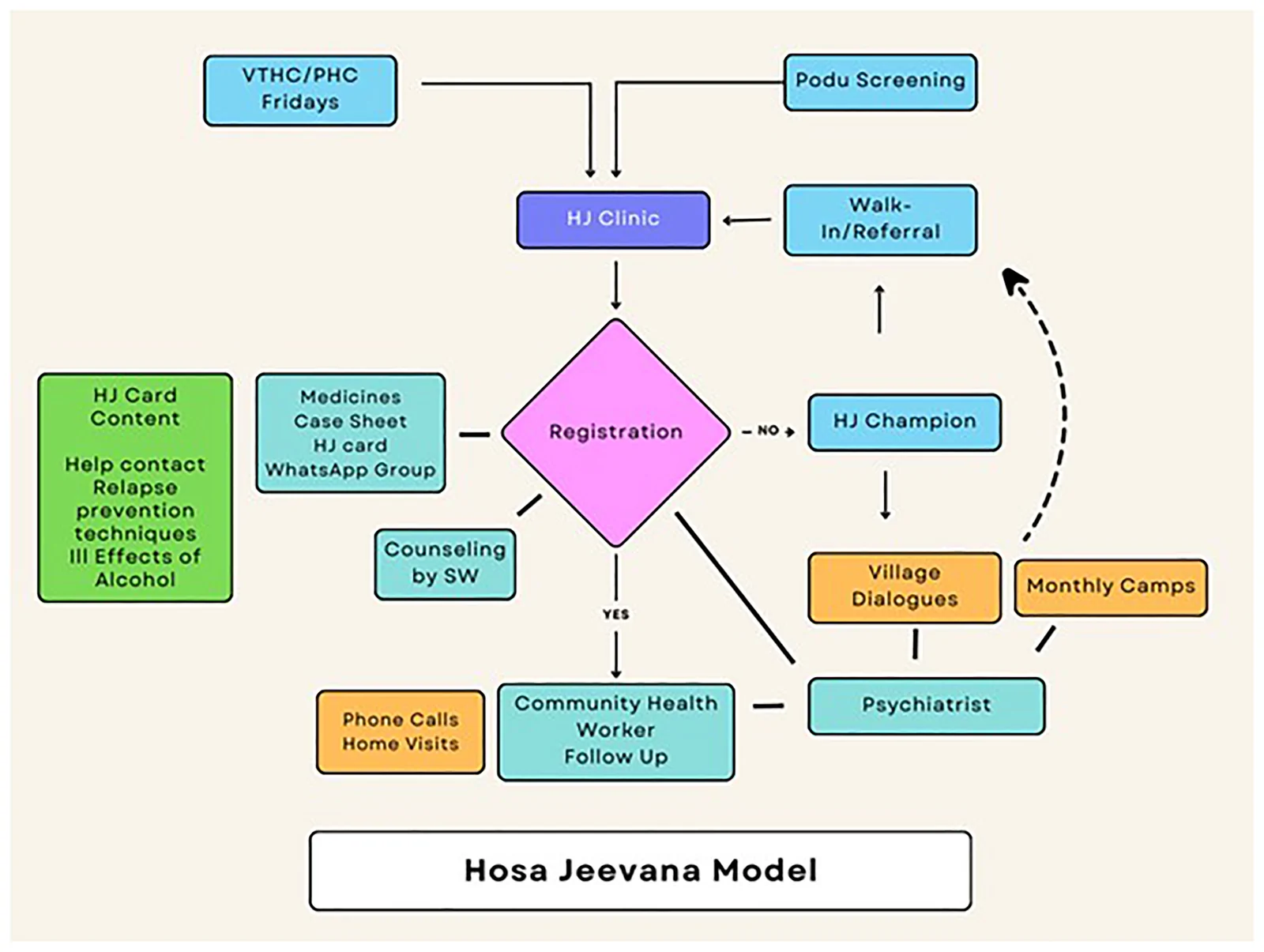
Hosa Jeevana program – model layout.

Through integrating clinical care with peer-led support, community outreach, and culturally appropriate education, the program aims to address addiction in a way that aligns with the lived realities of the Soliga people.

***Screening***

As a first step, community health workers from the tribal hospital and field-level workers from a CSR project in B.R. Hills initiated by the Institute of Public Health (IPH), Bengaluru, were trained to screen individuals using the ‘AUDIT’ tool (Alcohol Use Disorders Identification Test). The members of the HJ team translated the tool into the colloquial Kannada language spoken by the region’s inhabitants. Community health workers then refer individuals identified as having alcohol dependence (addiction) (cutoff score≥15) to the HJ clinic.

***Clinical component***

At the clinic, the clinical care coordinator compiles the screening sheets of persons identified with alcohol addiction and persons who are interested in the treatment. The model also works walk-ins into the clinic. They register those who consent to be part of the treatment program with an HJ identification number and open a case file at the hospital. The case file documents the medical history and other information as detailed in the data collection section of the methodology. The clinical care coordinator discusses the AUDIT score and medical history with the medical officer and plans the intervention. The medical officer prescribes the medication to the patient if required. If needed, the medical officer/clinical care coordinator consults the District Mental Health Programme psychiatrist. Based on the intervention planned, the clinical care coordinator counsels the patient and schedules follow-up visits to the clinic.

***Community component***

The community-level components of the intervention include monthly counselling, identifying HJ champions for champion-led meetings, quarterly podu (Soliga term for settlement/village) level interventions, culturally based IEC in the local language (using pamphlets, street plays and videos), and a WhatsApp patient support group.

**HJ Champions**

In the tribal settlements, if there are persons who have achieved sobriety for more than two years, the HJ program contacts them and requests that they support nearby persons interested in quitting alcohol use. If they are willing to help, the program calls these HJ champions and supports them in engaging with people in their respective settlements. They initiate discussions on alcohol addiction recovery, refer individuals interested in quitting alcohol use, and continue providing support to people regardless of their motivation to change.

**Community-level interventions**

In the community, the community health workers follow up with these patients and inform them to contact the HJ champion or clinical care coordinator for relevant support needed or at risk of relapse. The community health workers also coordinate health camps in the settlements. The respective HJ champion and doctor/psychiatrist will also be at these camps.

**IEC materials**

The community health workers also provide IEC materials designed in Kannada to the families screened for alcohol use and facilitate street plays co-designed with the local folk arts group on stories of mental health and alcohol addiction recovery.

**WhatsApp group**

Individuals receiving treatment are added to a WhatsApp support group if they express interest in real-time support and/or in contacting the clinical care coordinator in crisis, ensuring continuity of care.

The logic model of the Hosa Jeevana program ( Table 1) highlights the process indicators associated with the outputs and outcomes arising from these activities, which the researchers can study.

**Table 1.** Hosa Jeevana program – logic model layout.

Resources	Activities	Outputs	Possible Outcomes
Screening tools Medications IEC Materials Helpline Support group	Screening Hospital Referrals Brief Intervention Clinical consultation Phone follow-ups IEC distribution Village Dialogues Monthly camps	Individuals are screened, referred, and enrolled in the program. AUDIT score changes at baseline and follow-ups. Retention rates in treatment. Participation rates in peer support groups. Frequency of helpline usage and concerns raised Attendance and engagement rates in community sessions Thematic insights in stakeholder experiences Identification of barriers and facilitators influencing program adoption Culturally tailored educational materials for the Soliga community Documentation of the program delivery process Training & upskilling of local staff in delivering alcohol addiction support Reports on program feasibility and adoption	Change in alcohol use and related harms Increased access to culturally appropriate care Reduction in stigma Enhanced community participation Improved service delivery between the health center and the community-based program Evidence of the feasibility of program integration
**People** Clinical case coordinator Community health worker HJ Champion Medical officer Psychiatrist Nurse			

**Assumptions**

- Health workers can do the screening at their respective settlements. They receive funding for their work for the duration of the intervention.
- The medications from DMHP or the health centre are available on time. The psychiatrist is available for consultations.
- The IEC materials are available for health workers to distribute in the community and at events.
- The clinical case coordinator is available for dedicated phone support for the patients to contact and communicate for assistance and crisis intervention.
- Case files are updated, and documentation is done on time by the clinic case coordinator to interpret the data and find information on the indicators.

### Need for the study

While the current iteration of HJ incorporates elements of culturally sensitive care, it is unknown whether the Adivasi community in B.R. Hills has accepted and adopted the intervention. These researchers aim to address this critical gap by determining the acceptability and adoption of the intervention. In addition, by aiming to explore the subjective factors associated with acceptability and adoption, the study hopes to identify pathways for equitable and effective addiction care for the Soliga people living in B.R. Hills.

**What do we already know about this topic?**

Community-based interventions have shown effectiveness in improving addiction recovery outcomes by integrating peer support and culturally sensitive approaches. However, limited research exists on how these models can adapt to Indigenous tribal communities in India, where existing de-addiction services often fail to address cultural and structural barriers.

**What does this study add?**

This study evaluates the acceptability and adoption of a **culturally tailored addiction recovery program** for the Soliga tribal community. A formative evaluation approach with embedded mixed methods can provide empirical evidence on how community-driven models can enhance engagement and improve short-term recovery outcomes.

**How might this study affect research, practice, or policy?**

The findings will offer **actionable insights for designing and implementing culturally adapted de-addiction services** in tribal health programs. Policymakers and healthcare providers can use this evidence to develop **scalable, community-integrated addiction interventions**, improving accessibility and effectiveness in marginalised populations.

## Methodology

### Study setting

The intervention and, thereby, the study focus on the Soliga tribal community in the B. R. Hills region, focusing on individuals with alcohol addiction. The reach of the study includes:

❖   17 Soliga settlements where community-based interventions will be implemented.

❖   The Hosa Jeevana (HJ) clinic is central to clinical care and follow-ups.

❖   Community Champions and Health Workers engage with individuals in remote locations.

❖   Local healthcare institutions, mainly VHTC, integrate HJ interventions.

### Study design

The study, using an embedded mixed methods design, aims to determine the acceptability and adoption of the intervention, along with factors influencing them, in two phases:

a.   A quantitative phase that quantifies acceptability and adoption using clinical records of the patients enrolled in the HJ program.

b.   A qualitative phase will explore the facilitators and barriers to care-seeking that influence the acceptability and adoption of the intervention.

### Operational definitions

For this study, acceptability refers to how well the HJ program aligns with the cultural, social, and practical needs of the Soliga community. It is measured by community members’ and stakeholders’ attitudes toward the program, including their willingness to engage with it, their satisfaction with its components, and how well it addresses their cultural, social, and practical needs. This can be assessed through interviews with patients and multiple stakeholders.

Adoption in this study refers to how the HJ program is embraced and implemented within community and healthcare settings. It is measured by the degree of program integration into routine community practices, healthcare services, and the involvement of community members and healthcare providers in the program. Adoption indicators include the number of individuals utilising the program, the number of healthcare providers participating, and the extent to which the program becomes embedded within existing local infrastructures.

### Quantitative phase

***Study population and sampling***

○ Population: Patients with alcohol addiction enrolled on the HJ program from the 17 Soliga settlements.
○ Sampling: All consenting HJ program participants are enrolled between 2025 and 2026.

***Eligibility criteria***

Inclusion criteria: Soliga individuals with alcohol addiction above 18 years enrolled in the HJ program.

Exclusion criteria: Individuals with severe cognitive or psychiatric impairment that may hinder their ability to participate in an interview.

***Informed consent***

Informed consent will be obtained from all program participants before study enrolment and data collection. A written patient information sheet and informed consent form will be provided to the participants. Given the low literacy levels within the Soliga community, if the participants prefer an oral explanation, the clinic coordinator will clearly explain the study objectives, procedures, potential risks, and benefits in the local language. Participants will be assured their right to refuse or withdraw at any stage without impacting their access to services. The research team will document the written consent, and this approach has been reviewed and approved by the Institutional Ethics Committee (reference: IPH/24-25/E/304 for study 14/2024/FR).

***Data collection***

**Data collection tool – Clinical data**

We will derive data for the quantitative phase primarily from a customised clinical data collection form maintained at the HJ Clinic. The researcher has designed the form to capture longitudinal data for everyone enrolled in the intervention. Using this form will ensure consistency across records and data standardisation. The clinical data form will document information related to the following:

○ AUDIT scores at the baseline and 12 months following initial enrolment.
○ The **AUDIT** is an internationally validated screening tool for assessing alcohol use patterns and identifying at-risk individuals.
○ Medication adherence
○ Self-reported abstinence
○ Relapse episodes
○ Follow-up appointments
○ Follow-up contacts
○ Brief interventions
○ Consent to research
○ Demographic details

**Extracting data from the clinical data form**

The clinical care coordinator at the HJ clinic will be responsible for entering de-identified data from the clinical records into an electronic database that will be used to calculate indicators related to acceptability and adoption. The database will be maintained on a platform such as MS Excel.

**Data collection tool – Community-level and other data**

Routine documentation related to services provided at participation in community events and camps, and distribution of IEC material, will serve as the other information sources that will help calculate indicators.

**Data analysis**

The investigator will use free and open-source software (e.g. JAMOVI) to analyse the data gathered from various sources and measure acceptability and adoption using the following indicators.

### Qualitative phase

The qualitative phase of the study will focus on collecting data on barriers and facilitators for implementing the intervention, which will also help to identify the factors associated with acceptability and adoption. The qualitative phase will begin 9 months after the initial phase.

***Study population and sampling***

**Study population**

- To ensure sufficient representation, the study team will recruit participants (patients) from the HJ program, with different demographic characteristics and levels of engagement.
- Immediate family members of the participants
- HJ Champions
- Community health workers
- Clinical care coordinator

Sampling – Purposive and convenient sampling based on the study population group.

***Eligibility criteria***

**Patients (from the HJ program)**

- **Inclusion Criteria:**
  - Individuals who have **participated in the HJ program** (current or past).
  - Representation of diverse **demographics** (age, gender, socioeconomic status, etc.).
  - Varied levels of **engagement** (e.g., successful recovery, ongoing treatment, or disengaged).
- **Exclusion Criteria:**
  - Individuals with **severe cognitive or psychiatric impairment** that may hinder their ability to participate in an interview.

**Community Health Workers (CHWs)**

- **Inclusion Criteria:**
  - CHWs are involved in screening, referrals, and follow-ups related to the HJ program.
  - At least three months of experience working with the HJ program.
  - Familiarity with patient interactions and program delivery processes.
- **Exclusion Criteria:**
  - CHWs with limited or no direct involvement in the HJ program.

**HJ Champions**

- **Inclusion Criteria:**
  - Individuals formally designated as HJ champions by the program.
  - Active participation in program activities for at least three months.
  - Willingness to discuss their experiences engaging with the community, referring individuals, and supporting recovery.
- **Exclusion Criteria:**
  - Champions are no longer involved in the program or have disengaged for over two months.

**Clinical Care Coordinator**

- **Inclusion Criteria:**
  - The primary clinical care coordinator oversees the implementation of the HJ program.
  - Direct involvement in treatment delivery, referrals, and follow-ups.
- **Exclusion Criteria:**
  - Coordinator with limited or no direct patient interaction.

***Data collection***

**Data collection method and tools**

The data will be collected using audio-recorded interviews using interview guides, an audio recorder and transcription software (if possible).

The topics covered in the interviews will vary depending on the study population. The interviews will focus on understanding the barriers and facilitators to participating in or implementing the intervention from each stakeholder’s perspective.

The researcher will explore the following topics with patients in the intervention:

○ Reasons for seeking help.
○ Perceived benefits and challenges of the program.
○ Facilitators and barriers to care seeking.
○ Experience with healthcare providers and staff.
○ Perception of community-based activities and events

The researcher will explore the following topics with Community Health Workers:

○ Service Delivery Experiences
○ Perceived Effectiveness
○ Barriers and Facilitators to deliver the program
○ Collaboration and Support
○ Suggestions for Improvement

The researcher will explore the following topics with HJ Champions:

○ Role and Motivation
○ Experiences with referrals and support
○ Community engagement
○ Barriers and facilitators in supporting people
○ Sustainability and suggestions

The researcher will explore the following topics with the clinical care coordinator:

○ Program Coordination
○ Clinical service delivery
○ Program Adoption
○ Barriers and facilitators in implementation
○ Sustainability and Future Perspectives

**Data analysis**

The interviews will be recorded and transcribed verbatim. Other than transcripts, notes, reflections, and other observational data will be included during the analysis. The analysis will be done using QualCoder, following the principles of thematic analysis and combining pre-determined and emerging themes and sub-themes.

### Measuring acceptability and adoption

To quantify acceptability, we will use indicators related to acceptance of referrals, treatment initiation, engagement with the treatment, treatment retention and participation in community-level events. These indicators reflect how well the HJ program aligns with the cultural, social and practical needs of the Soliga community.

Indicators associated with the delivery of clinical services, HJ champion-related activities, and follow-ups will help measure adoption, reflecting how the program is integrated into community and healthcare settings. The specific indicators that will be used will be discussed later.

The researchers will calculate some indicators per treatment episode rather than at the individual level since we anticipate that some individuals will have more than one treatment episode. At the individual level, if there is a gap of 60 days between two clinical visits, the subsequent visit will be considered a new treatment episode.

***Measuring the success of the intervention***

While not the primary goal of this study, we plan to capture information related to the success of the intervention to inform future studies. We plan to use recovery as a success metric.

Recovery is a journey where a person works toward both overcoming alcohol use disorder (AUD) and quitting heavy drinking. It can also be seen as a destination — someone is considered “recovered” when they have successfully achieved and sustained freedom from AUD and heavy drinking over time. Currently, no agreed-upon definition of the term “recovery” exists in the alcohol treatment literature ^17^.

For this study, we will use one year as the time frame to measure the recovery metrics using the AUDIT score at recruitment as the baseline. We will also monitor if there is an increase in the gap between relapses, or, in other words, an increase in the duration of treatment between relapses.

***Indicators list***

The study team will use the following indicators to measure recovery from alcohol addiction:

➢   AUDIT Scores:

○ Baseline score at program entry
○ Follow-up assessments at six and 12 months post-enrolment
Reduction in AUDIT scores indicating decreased alcohol use

➢   Clinical Records:

○ Medication adherence and detoxification records.
○ Self-Reported Abstinence and Relapse Episodes: Participant-reported alcohol use frequency, triggers, and relapse occurrences data collected through structured interviews and patient follow-ups

The study team will measure the indicators shown in Table 2 to assess acceptability:

**Table 2.** Acceptability indicators and data sources.

Indicators	Data sources
Patient satisfaction with the Hosa Jeevana program	Patient interviews
Perceived cultural relevance of the intervention components (peer recruitment, culturally tailored materials and helpline support)	Patient interviews and family feedback
The willingness of patients to continue participating in the program	Program retention data from clinical records, patient interviews
Participants reported easy access to services (clinic visits, helpline use)	Clinic visits and helpline logs, patient interviews
Patient and family feedback on the program’s usefulness and cultural fit	Patient interviews and family feedback
Qualitative insights regarding their experiences with the intervention	Patient interviews
Retention rates over 6 and 12 months	Clinical records

The study team will measure the indicators shown in Table 3 to assess adoption:

**Table 3.** Adoption indicators and data sources.

Indicators	Data sources
Participation rates in HJ support groups	Attendance logs and interviews
Frequency of group meetings by community champions	Event logs and interviews with champions
Reach of IEC activities (pamphlets, street plays, videos)	Event records and community feedback
Community participation in IEC events and health camps	Event records and patient interviews
Engagement of HJ champions (sobriety achievements, referrals)	Champion tracking records and referral logs, interviews
Community Health Worker (CHW) involvement (follow-ups, referrals)	CHW tracking records and referrals, interviews
The program reaches across 17 Soliga settlements	Interview with clinical care coordinator

## Ethics and dissemination

The study has ethical approval (reference IPH/24-25/E/304 for study 14/2024/FR) from the Institutional Ethics Committee of the Institute of Public Health Bengaluru, the field-level institute that supervises the work of the principal investigator. Study results will be disseminated at national and international conferences and local Adivasi Sangha collaborative meetings and published in peer-reviewed journals and digital or print media.

Informed consent will include both phases and will be obtained for study enrolment. All interviews will be recorded with consent, and notes will be documented. Digital copies of notes will be stored on the office laptop. Recordings, notes, and observational checklists will be saved in an encrypted, password-protected folder on an office laptop or the cloud, with access restricted to the study team. Data shared with collaborators or for publication will remain anonymous. Non-participant observations, such as media-like photographs without individuals, will be included and tagged with qualitative data. Participants will be informed about data usage and their right to withdraw during the interview, ensuring they are not compelled to answer all questions. To protect anonymity, researchers will code identities with unique identifiers and omit personal information. After the project concludes, all data will be preserved for five years in a secure cabinet at the IPH Office, after which files will be deleted or securely destroyed.

## Results

The study team aims to demonstrate whether the HJ program is an acceptable and well-adopted intervention within the Soliga tribal community. The researchers will examine whether engagement with addiction recovery services increases by measuring clinic attendance, participation in peer support groups, and retention rates.

If the team identifies a significant reduction in AUDIT scores over time, indicating decreased alcohol consumption among participants, they can conduct further studies to explore scalability, monitoring, and evaluation. Additionally, qualitative interviews will offer a deeper understanding of the social, cultural, and logistical facilitators and barriers that influence care-seeking behaviours and treatment adherence. The team will also assess how community champions and culturally relevant materials influence engagement, leading to recommendations for scaling the intervention in other tribal communities.

### Limitations of the study

- Potential for selection bias in qualitative sampling.
- Challenges in generalising findings beyond the Soliga community.

## Discussion

There is growing evidence supporting the effectiveness of community-based interventions in addiction recovery, particularly those that integrate peer-led support and culturally sensitive approaches. Studies have shown that community-based programs can increase engagement and retention rates in addiction treatment by addressing stigma, accessibility, and cultural relevance ^18^. Additionally, research on Indigenous addiction recovery programs in Canada and Australia highlights that integrating traditional knowledge and peer mentorship can significantly improve long-term recovery outcomes ^14^. However, limited research exists on how such models can be adapted for tribal communities in India, making this study an essential contribution to the field.

Despite the effectiveness of community-driven addiction recovery models, most research remains focused on programmatic interventions rather than implementation frameworks. Ensuring that culturally adapted programs are sustainable and scalable requires understanding implementation science, which examines how interventions can be effectively integrated into existing healthcare systems ^19^. One of the primary challenges in implementing community-based addiction programs is the lack of institutional integration and long-term funding mechanisms, which can impact sustainability ^20^. By evaluating acceptability and adoption, this study will help identify key implementation factors for ensuring the success of the HJ program.

According to Proctor *et al.*, researchers can determine the success of an intervention by evaluating implementation outcomes such as acceptability, adoption, and feasibility ^19^. Measuring acceptability helps assess whether community members find the intervention culturally relevant and non-stigmatising, while adoption rates reflect how much individuals engage with available services. Conversely, feasibility examines how well healthcare providers can integrate the intervention into existing healthcare structures. By focusing on these constructs, this study will provide evidence-based insights into effectively implementing culturally sensitive addiction recovery models in tribal communities.

Ultimately, this research aims to inform policy and service delivery by generating evidence on how the HJ program can be scaled and sustained within public health systems. While India’s National Action Plan for Drug Demand Reduction emphasises rehabilitation and community-based care, it lacks specific tribal-focused interventions incorporating cultural perspectives. By demonstrating the impact of a peer-led, community-driven model, this study can potentially influence future tribal health policies and addiction recovery programs across India.

## Public-Private statement

Patients and caregivers will be involved in the refinement process of the intervention package. The researcher will inform the members of community collectives about the study. The researcher will share the findings with the participants and stakeholders.

## Data Availability

This manuscript outlines a study protocol. As the study is currently in the planning/implementation phase, no datasets have been generated or analysed. Upon completion of the study, de-identified data supporting the findings will be made openly available in a suitable public repository under a CC BY 4.0 license, subject to ethical approvals and participant consent. The Data Availability statement will be updated in future study results publications.

## References

[1] Tomar SP Kasar PK Tiwari R : Study of alcohol consumption and its sociodemographic determinants in a tribal village in Mandla district. *Int J Med Sci Public Health.* 2016;5(5):989–93. 10.5455/ijmsph.2016.10022016353

[2] Sadath A Jose K Meethal ST : Factors associated with alcohol misuse among Indigenous tribal men in Wayanad: a qualitative study. *Indian J Psychol Med.* 2019;41(6):516–22. 31772437 10.4103/IJPSYM.IJPSYM_326_19 PMC6875840

[3] Seshadri T Velho N Narasimhamurti NS : Examining tribal health inequalities around three forested sites in india: results of a cross-sectional survey. *J Family Med Prim Care.* 2020;9(9):4788–96. 33102260 10.4103/jfmpc.jfmpc_508_20 PMC7116252

[4] Rao VG Bhat J Yadav R : Smoking and alcohol consumption: risk factors for pulmonary tuberculosis among the tribal community in central India. *Indian J Tuberc.* 2017;64(1):40–3. 28166915 10.1016/j.ijtb.2016.11.009

[5] Pati S Chauhan AS Mahapatra P : Weaved into the cultural fabric: a qualitative exploration of alcohol consumption during pregnancy among tribal women in Odisha, India. *Subst Abuse Treat Prev Policy.* 2018;13(1):1–9. 29463287 10.1186/s13011-018-0146-5 PMC5819194

[6] Sadath A Jose K Jiji KM : Prevalence and determinants of substance use among Indigenous tribes in South India: findings from a tribal household survey. *J Racial Ethn Health Disparities.* 2022;9(1):356–66. 33495925 10.1007/s40615-021-00964-2

[7] Browne AJ Varcoe C Lavoie J : Enhancing health care equity with Indigenous populations: evidence-based strategies from an ethnographic study. *BMC Health Serv Res.* 2016;16(1): 544. 27716261 10.1186/s12913-016-1707-9 PMC5050637

[8] Prasad S Raushan R Tiwari R : Health status and healthcare care-seeking behavior: a study of tribal communities in Bijadandi block in Mandla district. *Int J Health Sci Res.* 2023;13(9). 10.52403/ijhsr.20230925

[9] Rowan M Poole N Shea B : Cultural interventions to treat addictions in Indigenous populations: findings from a scoping study. *Subst Abuse Treat Prev Policy.* 2014;9: 34. 25179797 10.1186/1747-597X-9-34 PMC4158387

[10] Betancourt JR Green AR Carrillo JE : Cultural competence in health care: emerging frameworks and practical approaches. New York: Commonwealth Fund, Quality of Care for Underserved Populations,2002;576. Reference Source

[11] Campinha-Bacote J : The process of cultural competence in the delivery of healthcare services: a model of care. *J Transcult Nurs.* 2002;13(3):181–4. 12113146 10.1177/10459602013003003

[12] Dale E Conigrave KM Kelly PJ : A Delphi yarn: applying Indigenous knowledges to enhance the cultural utility of SMART recovery Australia. *Addict Sci Clin Pract.* 2021;16(1):2. 33407873 10.1186/s13722-020-00212-8 PMC7787604

[13] Pandey M Clark M Beresh E : Wellness wheel clinics: a first nation community-partnered care model improving healthcare access, from healthcare providers’ perspectives. *Health Promot Int.* 2023;38(4): daad079. 37590386 10.1093/heapro/daad079

[14] Minkler M Wallerstein N : Community-based participatory research for health: from process to outcomes. 2nd ed. San Francisco: Jossey-Bass,2008. Reference Source

[15] World Health Organization: International standards for the treatment of drug use disorders: revised edition. Geneva: WHO,2020. Reference Source

[16] Rose A Minz S Manohari GP : Community perspectives on alcohol use among a tribal population in rural Southern India. *Natl Med J India.* 2015;28(3):117–21. 26724338

[17] Witkiewitz K Montes KS Schwebel FJ : What is recovery? *Alcohol Res.* 2020;40(3): arcr.v40.3.02. 32983748 10.35946/arcr.v40.3.01 PMC7505137

[18] Campbell C Jovchelovitch S : Health, community and development: towards a social psychology of participation. *J Community Appl Soc Psychol.* 2000;10(4):255–70. 10.1002/1099-1298(200007/08)10:4<255::AID-CASP582>3.0.CO;2-M

[19] Proctor E Silmere H Raghavan R : Outcomes for implementation research: conceptual distinctions, measurement challenges, and research agenda. *Adm Policy Ment Health.* 2011;38(2):65–76. 20957426 10.1007/s10488-010-0319-7 PMC3068522

[20] Guerrero EG Aarons GA Palinkas LA : Organisational capacity for service integration in community-based addiction health services. *Am J Public Health.* 2014;104(4):e40–7. 24524525 10.2105/AJPH.2013.301842 PMC4020143

