# Formative evaluation of an existing community-based intervention for alcohol addiction in the Soliga tribal community in Chamarajanagar, Karnataka

**DOI:** 10.12688/wellcomeopenres.24075.2

**Published:** 2026-10-22

**Authors:** Alen Chandy Alexander, Rahul Amruthapuri, Teddy Andrews Jaihind Jothikaran, Prashanth N. Srinivas

**Affiliations:** 1 Center for Training, Research & Innovation in Tribal Health, Institute of Public Health, Bengaluru, Karnataka, India; 2 Department of Social & Health Innovation, Prasanna School of Public Health, Manipal Academy of Higher Education, Manipal, Karnataka, India

**Keywords:** Alcohol addiction recovery, community-based intervention, Soliga tribe, mixed-methods study, culturally sensitive care, peer-led support, formative evaluation, implementation out-comes, short-term patient outcomes.

## Abstract

**Background:**

Socioeconomic stressors, cultural transitions, and limited access to healthcare often influence alcohol addiction among Indigenous communities. The Soliga tribal community lacks culturally tailored de-addiction services in the Chamarajanagar district of Karnataka, India. The Hosa Jeevana (HJ) program is a community-based intervention that integrates clinical care, peer-led support, and culturally relevant awareness strategies to support addiction recovery. This study protocol outlines the plan for evaluating the HJ program’s acceptability, adoption, and short-term patient outcomes through a formative approach.

**Study setting:**

The study will be conducted in the HJ clinic and 17 Soliga settlements in B.R. Hills, Karnataka, India, where the intervention is implemented.

**Method:**

This study will use an explanatory sequential mixed-methods design. In the first phase, clinical records, Alcohol Use Disorders Identification Test (AUDIT) scores, and program retention rates will be analysed to assess engagement, participation, and early indicators of recovery. In the second phase, in-depth interviews will explore facilitators and barriers to care-seeking and perceptions of program acceptability and adoption among Soliga individuals enrolled in the HJ program, HJ Champions (peer mentors), and other stakeholders such as community health workers and physicians. The qualitative findings will contextualise the trends observed in the quantitative analysis. Expected outcomes Implementation outcomes, i.e. acceptability, adoption and identification of barriers and facilitators of the program, are the expected outcomes of the study protocol.

**Discussion:**

The study findings will help refine the existing community-driven addiction recovery program and inform scaling up the intervention. While long-term sustainability falls outside the study’s scope, insights from this evaluation will lay the foundation for future research and policy integration.

## Plain language summary

Alcohol addiction is a significant health concern in many communities, including among Adivasi populations in southern Karnataka. However, existing healthcare services often struggle to reach and support individuals dealing with alcohol dependence effectively. This study aims to introduce and evaluate a culturally responsive intervention program called Hosa Jeevana (meaning "New Life"), explicitly designed for the Solega Adivasi people. The program offers support through health center-based services and community-based efforts. It includes mobile phone-based peer support groups, guidance from abstinent champions, awareness materials in local languages, and access to a 12-hour helpline staffed by an Adivasi counsellor.

The study will assess whether the program is acceptable and valuable to the community by tracking how well it is adopted and whether it helps people reduce or stop harmful alcohol use. By working alongside community members, the research aims to promote accessible, sustainable, and culturally appropriate care pathways, potentially shaping future health policies and services for Adivasi communities.

## Introduction

Research on alcohol use disorders (AUDs) among indigenous and tribal communities highlights structural and social determinants of health that worsen addiction. Researchers have found that socioeconomic stressors, cultural displacement, and limited healthcare access often contribute to alcohol use. ^1^ ^,^ ^2^ The incidence of tobacco and alcohol use in various tribal communities across India is twice as high as that in nearby non-tribal communities, ^3^ indicating the need for treatment options.

However, current de-addiction efforts in India primarily focus on urban or facility-based models, often failing to reach marginalised populations. More specifically, de-addiction and cessation services are yet to reach rural areas. ^4^ In addition, social determinants of health, like livelihood stress and social exclusion, negatively impact these communities, resulting in poorer treatment outcomes. The lack of context-specific public health programs further compounds this issue. ^5^ ^,^ ^6^ Research has demonstrated that generic addiction care models often fail to address the barriers faced by tribal populations, such as stigma, mistrust of formal healthcare systems, and a lack of culturally relevant materials and approaches. ^7^ ^,^ ^8^

According to global evidence, community-based addiction programs incorporating local cultural elements and peer support structures have demonstrated better outcomes than conventional clinical interventions. ^9^ Culturally sensitive care is healthcare, responsive to and respectful of the cultural beliefs, practices, and needs relevant to diverse patient populations. ^10^ It involves tailoring interventions to align with the target community’s social, linguistic, and cultural contexts. This approach improves health outcomes by fostering trust, enhancing patient-provider communication, and increasing the acceptability of healthcare services. ^11^

International models, such as Indigenous-led recovery programs in Canada and Australia, emphasise the role of peer champions and culturally appropriate interventions in addiction care. ^12^ ^,^ ^13^ Such approaches enhance the adoption and sustainability of interventions in tribal and marginalised populations. ^14^ The World Health Organisation (WHO) supports such models, noting that interventions that leverage community participation and local knowledge tend to yield higher engagement rates and long-term adherence to treatment. ^15^

The sociocultural contexts of tribal communities are not being well-characterised enough to design community-based interventions. ^16^ As a result, a significant research gap remains, and very limited empirical evidence exists on community-based, culturally sensitive de-addiction interventions in India, especially among Adivasi populations. In this paper, we describe how a team of healthcare staff and workers based in a tribal hospital and community settlements are implementing a clinic and community-based intervention package in B.R. Hills, Chamarajanagar district and present the plan for a formative evaluation of the intervention.

In this manuscript, we use the term *“tribal” and “Adivasi”* to refer to communities officially recognised as *Scheduled Tribes* under the Constitution of India. While we acknowledge that *“Indigenous”* is a preferred term in international discourse, *“tribal”* remains the term commonly used in Indian policy, legal, and programmatic contexts, including in health policy documents, census classification, and welfare schemes and *“Adivasi”* is the term commonly used by such populations in the South Indian context. This term reflects the legal identity and administrative categorisation under which these communities access state benefits and protections. We intend to use this term respectfully and contextually, aligning with how community members in this region and state institutions describe and engage with these populations.

### History of Hosa Jeevana

Hosa Jeevana did not originate from a single specific intervention development framework. Rather, the intervention evolved through iterative experiences in providing alcohol and tobacco-related care to members of the Soliga community. In its initial stages, the team did not draw on a formal evidence-based model or structured intervention-development method. Instead, development was guided by the needs and input expressed from community members, experiential knowledge of local practitioners, informal feedback, and observations of barriers to care and engagement. These experiences led the team to adapt the intervention’s location, delivery arrangements, and degree of community involvement over time. A program theory was subsequently developed when the team began planning the present formative evaluation to make the intervention’s assumptions and anticipated mechanisms explicit and to guide its assessment and further refinement.

Drawing on prior experience and responding to requests from the Soliga community for support in addressing substance use, one of the authors (PNS) established HJ in 2021 as a mental health and de-addiction clinic. The clinic was initially based at the Vivekananda Tribal Health Centre and served people from tribal settlements in the B. R. Hills region. Operating for half a day, it was jointly run by PNS and a community health worker, who screened attendees for mental health and substance use disorders and provided clinical care when indicated.

Subsequently, with the idea of improving the accessibility for the Soliga community members from other taluks and aligning it with the existing healthcare systems for deaddiction services, the clinic signed an MoU with a nearby taluk hospital and shifted to their campus. However, the team observed that the Taluk hospital staff, primarily from non-tribal backgrounds, did not take ownership of the clinic activities. The clinic appeared distant from the hospital’s existing services. The experiences of the Adivasi patients while approaching the hospital for deaddiction services reflected this distance.

As a result, the clinic returned to its earlier premises at the tribal hospital as a weekly clinic for clinical care, with the community health worker to follow up with the patients and clinician-led patient group meetings. There was a lack of consistency with patient attendance for the meetings. The team questioned whether the lack of attendance was due to a lack of interest in engaging, since the moderator was a clinician and not someone with a lived experience from the community with whom they could relate and share openly.

Reflecting on this, the team decided to reimagine the intervention as a community-driven intervention that seeks to provide a holistic model of alcohol addiction recovery.

### Redesigned Hosa Jeevana

The redesigned intervention contains clinical and community-level activities ( Figure 1). In redesigning HJ, culturally sensitive care was considered through discussions with stakeholders from the Soliga community, incorporating their inputs, traditions, and preferences.

**Figure 1.**
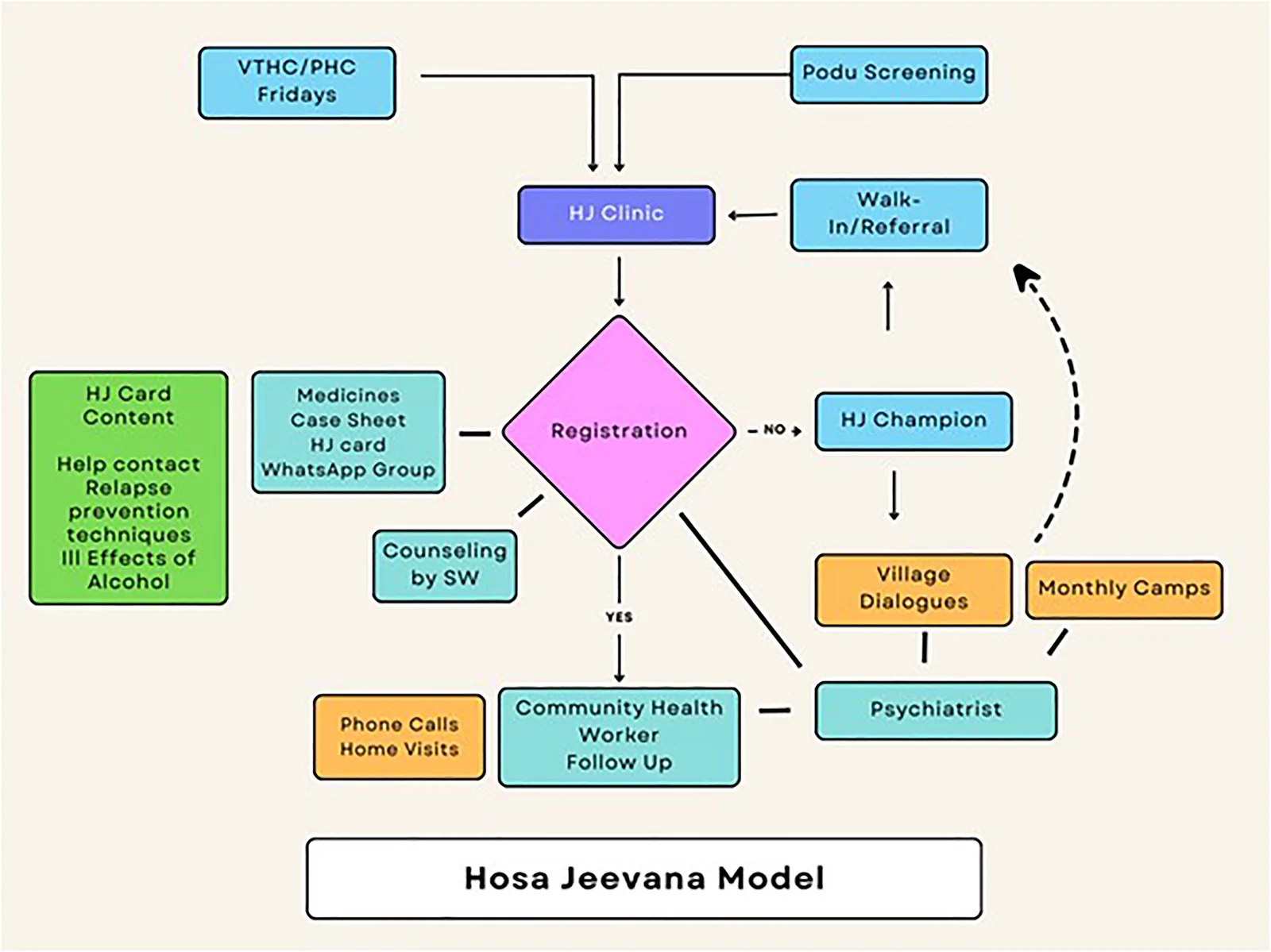
Hosa Jeevana program – model layout.

The decision to develop a multicomponent intervention was not based on evidence that the complete HJ package was superior to any single-component intervention. HJ initially operated primarily as a clinic-based service, and additional components were introduced iteratively in response to limitations observed during its delivery and to input from community members and practitioners. Clinical care alone could not address barriers related to geographical access, identification and referral in the community level, continuity of follow-up, culturally appropriate communication, or the need for support from people with relevant lived experience. The redesigned intervention combines components operating at the individual, clinical, and community levels, intended to address a distinct barrier identified during implementation. Although the individual components and underlying principles are supported by larger evidence on addiction care, peer support, culturally responsive interventions, and community engagement, evidence for the effectiveness of the combined HJ program in the Soliga context has not been determined. The present formative evaluation will assess how these components are accepted and adopted, how they work in practice, and which components need to be refined.

The culturally sensitive components of the intervention include:

• Support groups facilitated by community champions from the Soliga community.
• Culturally relevant educational materials, including visual and oral formats in the vernacular language.
• A focus on participatory methods to ensure that interventions resonate with community values.

Through integrating clinical care with peer-led support, community outreach, and culturally appropriate education, the program aims to address addiction in a way that aligns with the lived realities of the Soliga people.

***Screening***

As a first step, community health workers from the tribal hospital and field-level workers from a CSR project in B.R. Hills initiated by the Institute of Public Health (IPH), Bengaluru, were trained to screen individuals using the Alcohol Use Disorders Identification Test (AUDIT). The HJ team translated the tool into colloquial Kannada, the language spoken by the region’s inhabitants. Community health workers then refer individuals with AUDIT scores of 15 or higher to the HJ clinic for clinical assessment and appropriate care.

***Clinical component***

At the clinic, the clinical care coordinator compiles the screening sheets of persons identified with alcohol-related problems and those interested in treatment. The clinic also accepts walk-in patients. The clinical care coordinator registers those who consent to participate in the treatment program using an HJ identification number and opens a case file at the hospital. The case file documents the medical history and other information detailed in the data collection section of the methodology. The clinical care coordinator discusses the AUDIT score and medical history with the medical officer and plans the intervention. The medical officer prescribes medication if required. If needed, the medical officer or clinical care coordinator consults the District Mental Health Programme psychiatrist. Based on the planned intervention, the clinical care coordinator counsels the patient and schedules follow-up visits to the clinic.

***Community component***

The community-level components of the intervention include monthly counselling, identifying HJ Champions for champion-led meetings, quarterly podu (Soliga term for settlement/village) level interventions, culturally based IEC in the local language (using pamphlets, street plays and videos), and a WhatsApp patient support group.

**HJ Champions**

In the tribal settlements, if there are persons who have achieved sobriety for more than two years, the HJ program contacts them and requests that they support nearby persons interested in quitting alcohol use. If they are willing to help, the program calls these HJ Champions and supports them in engaging with people in their respective settlements. They initiate discussions on alcohol addiction recovery, refer individuals interested in quitting alcohol use, and continue providing support to people regardless of their motivation to change.

**Community-level interventions**

In the community, community health workers follow up with patients and advise them to contact the HJ Champion or clinical care coordinator when they need support or are at risk of relapse. Community health workers also coordinate health camps in the settlements. The relevant HJ Champion and doctor or psychiatrist may also attend these camps.

**IEC materials**

The community health workers also provide IEC materials designed in Kannada to the families screened for alcohol use and facilitate street plays co-designed with the local folk arts group on stories of mental health and alcohol addiction recovery.

**WhatsApp group**

Individuals receiving treatment are added to a WhatsApp support group if they express interest in real-time support and/or in contacting the clinical care coordinator in crisis, ensuring continuity of care.

**Integration with existing systems of care**

HJ is designed to connect community-based identification and support with clinical care through the tribal hospital, rather than to operate solely as a stand-alone addiction or mental health service. Within the formal public health system, primary healthcare staff are expected to screen for and identify people requiring de-addiction care and refer them to the District Mental Health Programme (DMHP). However, the experiences informing HJ suggested that access through this pathway was limited by inconsistent identification and referral at the primary-care level, as well as by the geographical distance between Soliga settlements and available services. These observations informed the development of a more locally accessible pathway involving community health workers, HJ Champions, the tribal hospital, and the HJ clinical care coordinator.

The community health workers and HJ champions can identify and refer individuals who may require support, while individuals may also attend the HJ clinic directly. At the clinic, the clinical care coordinator and medical officer review screening and clinical information, initiate or coordinate treatment, and seek input from the DMHP psychiatrist when required. Community follow-up, settlement-level camps, telephone support, and the WhatsApp group are intended to maintain continuity between the clinic and the community. HJ therefore serves as a partial bridge between Soliga settlements and existing mental health and de-addiction services.

Screening information is compiled by the clinical care coordinator, and individuals enrolled in the program receive an HJ identification number and a hospital case file. Clinical and follow-up information is recorded in the case file, and customised HJ records, with de-identified information subsequently entered into a separate electronic database for the formative evaluation. Although these arrangements provide functional links between community and clinical care, HJ referral and data flows are not yet fully embedded within routine public-sector referral and health-information systems. The evaluation will examine how these links operate in practice and identify opportunities to strengthen primary-care identification, two way directional referrals, referral feedback, shared documentation, and continuity of care.

The logic model of the Hosa Jeevana program ( Table 1) highlights the process indicators associated with the outputs and outcomes arising from these activities, which the researchers can study.

**Table 1.** Hosa Jeevana program – logic model layout.

Resources	Activities	Outputs	Possible Outcomes
Screening tools Medications IEC Materials Helpline Support group **People** Clinical case coordinator Community health worker HJ Champion Medical officer Psychiatrist Nurse	Screening Hospital Referrals Brief Intervention Clinical consultation Phone follow-ups IEC distribution Village Dialogues Monthly camps	Individuals are screened, referred, and enrolled in the program. AUDIT score changes at baseline and follow-ups. Retention rates in treatment. Participation rates in peer support groups. Frequency of helpline usage and concerns raised. Attendance and engagement rates in community sessions. Thematic insights into stakeholder experiences. Identification of barriers and facilitators influencing program adoption. Culturally tailored educational materials for the Soliga community. Documentation of the program delivery process. Training and upskilling of local staff in delivering alcohol-addiction support. Reports on program acceptability, adoption, and implementation barriers and facilitators.	Change in alcohol use and related harms. Increased access to culturally appropriate care. Reduction in stigma. Enhanced community participation. Improved service delivery between the health centre and the community-based program. Evidence relevant to program integration and potential sustainability.

**Assumptions**

• Health workers can do the screening at their respective settlements. They receive funding for their work for the duration of the intervention.
• The medications from DMHP or the health centre are available on time. The psychiatrist is available for consultations.
• The IEC materials are available for health workers to distribute in the community and at events.
• The clinical care coordinator is available for dedicated phone support for the patients to contact and communicate for assistance and crisis intervention.
• Case files are updated, and documentation is done on time by the clinic case coordinator to interpret the data and find information on the indicators.

### Need for the study

While the current iteration of HJ incorporates elements of culturally sensitive care, it is unknown whether the Adivasi community in B.R. Hills has accepted and adopted the intervention. These researchers aim to address this critical gap by determining the acceptability and adoption of the intervention. In addition, by aiming to explore the subjective factors associated with acceptability and adoption, the study hopes to identify pathways for equitable and effective addiction care for the Soliga people living in B.R. Hills.

### What do we already know about this topic?

Global evidence shows effectiveness in community-based interventions in improving addiction recovery outcomes by integrating peer support and culturally sensitive approaches. However, limited research exists on how these models can adapt to indigenous and tribal communities in India, where cultural and structural barriers are not addressed in existing de-addiction services.

### What does this study add?

This study evaluates the acceptability and adoption of a culturally tailored addiction recovery program for the Soliga tribal community. A formative evaluation using an explanatory sequential mixed-methods design can provide empirical evidence on patterns of engagement and short-term recovery outcomes, and use qualitative findings to explain the factors influencing these patterns.

### How might this study affect research, practice, or policy?

The findings will offer insights actionable for designing and implementing culturally adapted de-addiction services in tribal health programs. Policymakers and healthcare providers can use this evidence to develop scalable, community-integrated addiction interventions, improving accessibility and effectiveness in marginalised populations.

## Methodology

### Study setting

The intervention and, thereby, the study focus on the Soliga tribal community in the B. R. Hills region, focusing on individuals with alcohol addiction. The reach of the study includes:

• 17 Soliga settlements where community-based interventions will be implemented.
• The Hosa Jeevana (HJ) clinic is central to clinical care and follow-ups.
• Community Champions and Health Workers engage with individuals in remote locations.
• Local healthcare institutions, mainly VHTC, integrate HJ interventions.

### Study design

This study will use an explanatory sequential mixed methods design to examine the acceptability and adoption of the intervention and the factors influencing these outcomes. The study will proceed in two linked phases:

a. In the first phase, quantitative data from clinical and program records will be analysed to describe patterns in referral acceptance, treatment initiation, engagement, retention, follow-up, and participation in community-level activities.
b. In the second phase, qualitative interviews will explore the barriers, facilitators, and contextual factors that may explain the quantitative patterns. Preliminary quantitative findings will inform purposive selection of participants with varied levels of program engagement and may be used to refine the emphasis of the interview guides.

### Operational definitions

In implementation science, acceptability is defined as the perception among implementation stakeholders that an intervention is agreeable, palatable, or satisfactory. ^19^ In this study, acceptability refers to the perceptions of HJ among patients, adult immediate family members, HJ Champions, healthcare providers, and the clinical care coordinator. It includes their views on the intervention’s cultural and social responsiveness, perceived usefulness, burden, comfort, and satisfaction with its components. These perceptions will be explored qualitatively. Referral acceptance, initiation of treatment, continued engagement, retention, and participation in community activities will be treated as complementary behavioural indicators that may support the assessment of acceptability but cannot be interpreted as direct measures of stakeholder perceptions.

In implementation science, adoption is defined as the intention, initial decision, or action to try or employ an intervention. ^19^ In this study, adoption refers to the uptake and use of HJ components by community members, HJ Champions, healthcare providers, and the participating healthcare institution. It will be assessed through referrals to HJ, delivery of clinical services, participation by healthcare providers, HJ Champion activities, follow-up contacts, and delivery of community-level activities. Qualitative interviews will explore the factors influencing decisions to initiate or participate in these activities. The extent to which HJ becomes integrated into the routine community and healthcare infrastructure will be considered a related indicator of institutional embedding rather than adoption alone.

### Quantitative phase

***Study population and sampling***

• Population: Patients enrolled in the HJ program for alcohol addiction care across the 17 Soliga settlements.
• Sampling: All consenting HJ program participants enrolled within a window of 12 months will be included.

***Eligibility criteria***

• Inclusion criteria: Soliga individuals aged 18 years and above who are enrolled in HJ for alcohol addiction care.
• Exclusion criteria: Individuals unable or unwilling to provide informed consent for study participation.

***Informed consent***

Informed consent will be obtained from all program participants before study enrolment and data collection. A written patient information sheet and informed consent form will be provided to the participants. Given the low literacy levels within the Soliga community, if the participants prefer an oral explanation, the clinic coordinator will clearly explain the study objectives, procedures, potential risks, and benefits in the vernacular. Participants will be assured their right to refuse or withdraw at any stage without impacting their access to services. The research team will document the written consent, and this approach has been reviewed and approved by the Institutional Ethics Committee (reference: IPH/24–25/E/304 for study 14/2024/FR).

***Data collection***

**Data collection tool – Clinical data**

We will derive data for the quantitative phase primarily from a customised clinical data collection form maintained at the HJ Clinic. The researcher has designed the form to capture longitudinal data for everyone enrolled in the intervention. Using this form will ensure consistency across records and data standardisation. The clinical data form will document information related to the following:

• AUDIT scores at baseline and at six and 12 months following initial enrolment.

The AUDIT is internationally validated and has demonstrated acceptable psychometric properties in Indian populations. The HJ team translated and adapted the tool into the colloquial Kannada that Soliga participants routinely use when communicating with health workers. Trained community health workers will administer this version orally when participants are unable to read or prefer oral administration, and the same version will be used at baseline and follow-up. The psychometric properties of this locally adapted version have not been independently established. The other quantitative indicators will be extracted from clinical and program records and do not involve the administration of additional standardised scales.

• Medication adherence
• Self-reported abstinence
• Relapse episodes
• Follow-up appointments
• Follow-up contacts
• Brief interventions
• Consent to research
• Demographic details

**Extracting data from the clinical data form**

The clinical care coordinator at the HJ clinic will be responsible for entering de-identified data from the clinical records into an electronic database that will be used to calculate indicators related to acceptability and adoption. The database will be maintained on a platform such as MS Excel.

**Data collection tool – Community-level and other data**

Routine documentation related to services provided, participation in community events and camps, and the distribution of IEC material will serve as additional information sources to help calculate indicators.

**Data analysis**

Quantitative indicators derived from clinical records, attendance and referral logs, and routine program documentation will be analysed using JAMOVI, a free and open-source statistical software. Descriptive statistics will be used to summarise referral acceptance, treatment initiation, engagement and retention, participation in community activities, and the involvement of HJ Champions and healthcare providers. Tables 2 and 3 present the set of acceptability and adoption indicators, along with their corresponding quantitative and qualitative data sources. Interview-derived data will be analysed thematically, as described in the Qualitative Phase, and integrated with the quantitative findings during the mixed-methods interpretation.

**Table 2.** Acceptability indicators and data sources.

Indicators	Data sources
Patient satisfaction with the Hosa Jeevana program	Patient interviews
Perceived cultural relevance of the intervention components (peer recruitment, culturally tailored materials and helpline support)	Patient interviews and family feedback
The willingness of patients to continue participating in the program	Program retention data from clinical records, patient interviews
Participants reported easy access to services (clinic visits, helpline use)	Clinic visits and helpline logs, patient interviews
Patient and family feedback on the program’s usefulness and cultural fit	Patient interviews and family feedback
Qualitative insights regarding their experiences with the intervention	Patient interviews
Retention rates over 6 and 12 months	Clinical records

**Table 3.** Adoption indicators and data sources.

Indicators	Data sources
Participation rates in HJ support groups	Attendance logs and interviews
Frequency of group meetings led by HJ Champions	Event logs and interviews with HJ Champions
Reach of IEC activities (pamphlets, street plays, videos)	Event records and community feedback
Community participation in IEC events and health camps	Event records and participant interviews
Engagement of HJ Champions, including peer support and referrals	Champion tracking records and referral logs, interviews
Healthcare-provider involvement in screening, clinical care, follow-up, and referral	Clinical, screening, referral, and follow-up records; healthcare-provider interviews
Number and proportion of the 17 Soliga settlements reached by HJ activities	Program activity records and interview with the clinical care coordinator

### Qualitative phase

The qualitative phase will begin approximately nine months after the quantitative phase commences, allowing sufficient program data to accumulate for preliminary analysis. These preliminary quantitative findings will inform purposive selection of participants with varied experiences and levels of engagement and may be used to refine the emphasis of the interview guides. The interviews will explore barriers, facilitators, and contextual factors that may explain observed patterns in acceptability and adoption. Quantitative data collection may continue while the qualitative data are collected and analysed.

***Study population and sampling***

**Study population**

The qualitative study population will include current HJ participants (patients), adult immediate family members of current participants, HJ Champions, healthcare providers involved in HJ, including community health workers and medical officers, and the clinical care coordinator.

**Sampling**

Participants will be purposively selected to capture variation in demographic characteristics, treatment experiences, levels of program engagement, and implementation roles. The anticipated sample will include approximately 12–15 current HJ participants, 8–10 adult immediate family members, 3–5 healthcare providers, 5–8 HJ Champions, and the clinical care coordinator, giving an estimated total sample of 29–39 stakeholders, subject to consent and availability. Healthcare providers may include community health workers, nurses and medical officers involved in HJ.

Data collection and preliminary analysis will proceed iteratively. For patients, adult immediate family members, and healthcare providers, interviews will continue within the anticipated ranges until collection reaches thematic saturation. More participants may be recruited if further thematic depth is required and feasible. Five to eight HJ Champions will be purposively selected based on accessibility at the time of interview planning and variation in program participation. Their differing levels of participation may provide insights into capacity-building needs. The clinical care coordinator will be included as a key informant. The final sample size may therefore vary from the anticipated total.

***Eligibility criteria***

**Patients**

• **Inclusion Criteria:**
  o Aged 18 years or older and currently participating in the HJ program.
  o Able to communicate sufficiently in colloquial Kannada to participate in an interview and able and willing to provide informed consent.
  o Purposively selected to reflect variation in demographic characteristics, treatment experiences, and levels of program engagement.
• **Exclusion Criteria:**
  o Experiencing acute intoxication or withdrawal at the time of the interview; unable to provide informed consent because of severe cognitive or psychiatric impairment; or too medically or psychologically unwell to participate safely at the time of the interview.

**Adult immediate family members**

• **Inclusion criteria:**
  o Aged 18 years or older.
  o Identified by a current HJ participant as an immediate family member who is involved in or familiar with the participant’s treatment, support, or recovery experience.
  o Able to communicate sufficiently in colloquial Kannada to participate in an interview.
  o Able and willing to provide informed consent. The HJ participant must also have agreed to the family member being contacted.
• **Exclusion criteria:**
  o Insufficient knowledge of the participant’s engagement with HJ to discuss the program; unable to provide informed consent; or participation is considered likely to create significant distress, family conflict, or a risk to the HJ participant’s confidentiality.

**Healthcare providers**

• **Inclusion Criteria:**
  o Community health workers or medical officers involved in HJ screening, referrals, follow-up, clinical assessment, or treatment delivery.
  o At least three months of experience working with the HJ program.
  o Familiarity with patient care or program delivery processes.
• **Exclusion Criteria:**
  o Healthcare providers who are unwilling to provide informed consent.

**HJ Champions**

• **Inclusion Criteria:**
  o Individuals currently designated as HJ Champions by the program.
  o HJ Champions will be eligible regardless of the duration or current level of their participation in program activities.
  o Able and willing to provide informed consent and discuss their experience of the role, including any support or capacity-building needs.
• **Exclusion Criteria:**
  o HJ Champions who are unwilling to provide informed consent.

**Clinical Care Coordinator**

• **Inclusion Criteria:**
  o The primary clinical care coordinator oversees the implementation of the HJ program.
  o Direct involvement in treatment delivery, referrals, and follow-ups.
• **Exclusion Criteria:**
  o The clinical care coordinator will be excluded only if unwilling to provide informed consent.

***Data collection***

**Data collection method and tools**

Data will be collected through audio-recorded, semi-structured interviews using stakeholder-specific interview guides. Audio recordings will be transcribed using transcription software where feasible and checked against the recordings for accuracy.

The interview guides will undergo expert content review by individuals with relevant expertise in qualitative research, implementation research, addiction or mental health care, and tribal or community health. The reviewers will assess the relevance, clarity, comprehensiveness, and cultural appropriateness of the questions. The guides will be revised in response to this review and may be further refined based on preliminary quantitative findings and early interview experience.

Interviewers will be trained to recognise and respond to participant distress. If a participant reports suicidal thoughts or other indications of immediate risk, the interview will be paused, confidentiality may be breached to the extent required to protect the participant, and the clinical care coordinator or medical officer will be contacted for urgent assessment and referral.

The topics covered in the interviews will vary depending on the study population. The interviews will focus on understanding the barriers and facilitators to participating in or implementing the intervention from each stakeholder’s perspective.

The researcher will explore the following topics with patients in the intervention:

• Reasons for seeking help.
• Perceived benefits and challenges of the program.
• Facilitators and barriers to care seeking.
• Experience with healthcare providers and staff.
• Perception of community-based activities and events

The researcher will explore the following topics with adult immediate family members:

• Their experience of supporting the participant
• Perceived effects of alcohol use and treatment on the family
• Barriers and facilitators to treatment engagement
• Perceived usefulness and cultural fit of HJ
• Suggestions for improving family and community support.

The researcher will explore the following topics with healthcare providers:

• Service Delivery Experiences
• Perceived Effectiveness
• Barriers and Facilitators to deliver the program
• Collaboration and Support
• Suggestions for Improvement

The researcher will explore the following topics with HJ Champions:

• Role and Motivation
• Experiences with referrals and support
• Community engagement
• Barriers and facilitators in supporting people
• Sustainability and suggestions

The researcher will explore the following topics with the clinical care coordinator:

• Program Coordination
• Clinical service delivery
• Program Adoption
• Barriers and facilitators in implementation
• Sustainability and Future Perspectives

**Data analysis**

The interviews will be recorded and transcribed verbatim. In addition to transcripts, notes, reflections, and other observational data will be included in the analysis. The analysis will be conducted using QualCoder, following the principles of thematic analysis and combining predetermined and emerging themes and sub-themes.

**Mixed methods integration**

Integration will occur at three stages. First, preliminary quantitative findings will inform purposive selection of qualitative participants with varied levels of program engagement. Second, the quantitative findings may be used to refine the emphasis of the qualitative interviews so that observed patterns can be explored further. Third, after the quantitative and qualitative data have been analysed separately, quantitative indicators and qualitative themes will be compared using an integration matrix. The research team will examine areas of convergence, complementarity, and divergence to develop an integrated explanation of the intervention’s acceptability and adoption and to identify areas for refinement.

### Measuring acceptability and adoption

Acceptability will be assessed primarily through stakeholder accounts of whether HJ and its components are satisfactory, comfortable, useful, and responsive to the cultural, social, and practical context of the Soliga community. Referral acceptance, treatment initiation, engagement, retention, and participation in community-level activities will be used as complementary behavioural indicators. These indicators may suggest willingness to engage with HJ but will not, by themselves, be interpreted as direct measures of perceived acceptability.

Adoption will be assessed through the uptake and use of HJ by the individuals and settings expected to deliver or participate in it. Indicators will include healthcare-provider participation, clinical service delivery, referrals and follow-ups by healthcare providers, activities conducted by HJ Champions, and delivery of community-level activities. Qualitative findings will be used to explain the decisions and contextual factors influencing adoption. Indicators concerning routine integration and institutional embedding will be interpreted separately as evidence relevant to the program’s penetration and potential sustainability. The complete indicators and data sources are presented in Tables 2 and 3.

The researchers will calculate some indicators per treatment episode rather than at the individual level since we anticipate that some individuals will have more than one treatment episode. At the individual level, if there is a gap of 60 days between two clinical visits, the subsequent visit will be considered a new treatment episode.

***Exploratory recovery indicators***

Although recovery is not a primary outcome of this formative evaluation, the study will capture exploratory clinical and alcohol-use indicators to inform future outcome studies.

Recovery is a journey where a person works toward both overcoming alcohol use disorder (AUD) and quitting heavy drinking. It can also be seen as a destination: someone is considered “recovered” when they have successfully achieved and sustained freedom from AUD and heavy drinking over time. Currently, no agreed-upon definition of the term “recovery” exists in the alcohol treatment literature. ^17^

For this study, we will use one year as the time frame to measure the recovery metrics using the AUDIT score at recruitment as the baseline. We will also monitor whether the interval between relapses increases over time. Broader dimensions of personal, social, and functional recovery will not be measured using a standardised recovery instrument in this formative evaluation and may be examined using a culturally adapted measure in a future outcome-evaluation study.

***Indicators list***

The study team will use the following indicators to measure recovery from alcohol addiction:

AUDIT scores:

• Baseline score at program entry
• Follow-up assessments at six and 12 months post-enrolment
• Reduction in AUDIT scores indicating decreased alcohol use

Clinical records:

• Medication adherence and detoxification records
• Self-reported abstinence and relapse episodes, including participant-reported alcohol-use frequency, triggers, and relapse occurrences documented through structured interviews and patient follow-ups

The study team will measure the indicators shown in Table 2 to assess acceptability:

The study team will measure the indicators shown in Table 3 to assess adoption:

## Ethics and dissemination

The study has ethical approval (reference IPH/24–25/E/304 for study 14/2024/FR) from the Institutional Ethics Committee of the Institute of Public Health Bengaluru, the field-level institute that supervises the work of the principal investigator. Study results will be disseminated at national and international conferences and local Adivasi Sangha collaborative meetings and published in peer-reviewed journals and digital or print media.

Consent procedures will cover the study activities applicable to each participant group. For quantitative participants, consent will include the use of clinical and program data for the evaluation; for qualitative participants, separate consent will be obtained for the interview and audio recording. Notes and observational data will be documented as applicable. Digital files, including recordings, notes, and observational checklists, will be stored in encrypted, password-protected folders on an office laptop and/or an institution-approved secure cloud service, with access restricted to authorised study-team members. Physical consent forms and other hard-copy records will be stored in a locked cabinet at the IPH office. Data shared with collaborators or used in reports and publications will be de-identified. Non-participant observations, such as photographs that do not include identifiable individuals, may be linked to the qualitative data. Participants will be informed about data use, their right to decline individual questions, and their right to withdraw from the interview. Participant confidentiality will be protected by assigning unique study identification codes and removing direct personal identifiers from research records and analysis datasets. Any document linking participant identities to study identification codes will be stored separately and securely. Data will be retained for five years after the project concludes, after which digital files will be securely deleted and physical records securely destroyed.

## Expected results

The study will describe the acceptability and adoption of the HJ program within the Soliga community. It will examine patterns in referral acceptance, treatment initiation, clinic attendance, participation in peer-support and community activities, and retention in care.

Changes in AUDIT scores and other clinical indicators over follow-up will be reported as exploratory alcohol-use outcomes rather than as evidence of intervention effectiveness. Qualitative interviews will provide a deeper understanding of the social, cultural, and logistical facilitators and barriers that influence care-seeking, engagement, and treatment adherence. The findings will identify potential refinements to HJ and considerations for future outcome evaluation, sustainability, and scale-up.

### Limitations of the study

This study has several limitations. Purposive and accessibility-based qualitative sampling may introduce selection bias and will not capture the experiences of people who have left HJ. The colloquial Kannada version of the AUDIT has not undergone independent psychometric validation. Routine records may be incomplete or inconsistent, and self-reported alcohol use may be affected by recall and social-desirability bias. Recovery will be assessed using exploratory clinical and alcohol-use indicators rather than a validated multidimensional recovery measure. Finally, the absence of a comparison group and the formative design preclude causal conclusions about effectiveness, and the findings may not generalise beyond the Soliga context.

## Discussion

Evidence suggests that community-based approaches may support engagement in addiction care, particularly when they incorporate peer support, attention to access barriers, and culturally responsive practices 18. Research on Indigenous addiction recovery programs in Canada and Australia also indicates that traditional knowledge and peer mentorship may improve the relevance and accessibility of care 14. However, evidence on how multicomponent models operate in tribal communities in India remains limited. This study will contribute context-specific implementation evidence rather than test the effectiveness of the HJ program.

Although community-driven addiction recovery models show promise, much of the literature focuses on program delivery or outcomes rather than how such interventions are implemented in routine systems. Understanding implementation is important for assessing how culturally adapted programs can be integrated into existing healthcare structures and what may support their longer-term sustainability. ^19^ Institutional integration and dependable funding remain challenges for community-based addiction services. ^20^ By evaluating acceptability and adoption, this study will identify implementation barriers, facilitators, and areas for refinement within HJ.

Proctor et al. describe acceptability and adoption as distinct implementation outcomes. ^19^ Acceptability concerns stakeholders’ perceptions that an intervention is agreeable, satisfactory, or appropriate to their experience, whereas adoption concerns the intention, decision, or action to initiate or use an intervention. In this study, qualitative accounts will be central to understanding acceptability, while service uptake, provider participation, referrals, HJ Champion activities, and delivery of community components will inform adoption. Feasibility and sustainability are relevant to interpretation but are not specified as primary implementation outcomes in this formative evaluation.

This study has several strengths. It evaluates an existing, community-based program in a setting where evidence on culturally responsive addiction care remains limited. The mixed-methods design combines routine clinical and program data with in-depth accounts from participants, HJ Champions, community health workers, and clinicians, allowing quantitative patterns to be interpreted in their social and cultural context. The explicit focus on acceptability and adoption can generate practical evidence for refining service delivery, while the involvement of community-based stakeholders strengthens the contextual relevance of the evaluation.

This research is intended to inform refinement of HJ and future decisions about service integration, evaluation, and potential scale-up. India’s National Action Plan for Drug Demand Reduction emphasises rehabilitation and community-based care but provides limited guidance on culturally responsive approaches for tribal communities. By documenting how a peer-led, community-driven model is accepted, adopted, and implemented in the Soliga context, the study may offer lessons for future tribal health policy and addiction-service development. Further outcome and comparative research would be required before drawing conclusions about effectiveness or transferability to other settings.

## Patient and public involvement statement

Patients and caregivers will be involved in the refinement process of the intervention package. The researcher will inform the members of community collectives about the study. The researcher will share the findings with the participants and stakeholders.

## Data Availability

This manuscript reports a study protocol and does not present or analyse study findings. Since the study involves a small Adivasi community and potentially sensitive clinical and qualitative information, participant-level data will not be made openly available where there is a risk of re-identification. De-identified quantitative data and carefully reviewed qualitative material may be shared upon reasonable request, subject to participant consent, ethical approval, and applicable institutional data-governance requirements. The data availability statement will be updated in publications reporting the study findings.

## References

[1] Tomar SP Kasar PK Tiwari R : Study of alcohol consumption and its sociodemographic determinants in a tribal village in Mandla district. *Int J Med Sci Public Health.* 2016;5(5):989–993. 10.5455/ijmsph.2016.10022016353

[2] Sadath A Jose K Meethal ST : Factors associated with alcohol misuse among Indigenous tribal men in Wayanad: a qualitative study. *Indian.* *J Psychol Med.* 2019;41(6):516–522. 31772437 10.4103/IJPSYM.IJPSYM_326_19 PMC6875840

[3] Seshadri T Velho N Narasimhamurti NS : Examining tribal health inequalities around three forested sites in india: results of a cross-sectional survey. *J Family Med Prim Care.* 2020;9(9):4788–4796. 33102260 10.4103/jfmpc.jfmpc_508_20 PMC7116252

[4] Rao VG Bhat J Yadav R : Smoking and alcohol consumption: risk factors for pulmonary tuberculosis among the tribal community in central India. *Indian J Tuberc.* 2017;64(1):40–43. 28166915 10.1016/j.ijtb.2016.11.009

[5] Pati S Chauhan AS Mahapatra P : Weaved into the cultural fabric: a qualitative exploration of alcohol consumption during pregnancy among tribal women in Odisha, India. *Subst Abuse Treat Prev Policy.* 2018;13(1):1–9. 29463287 10.1186/s13011-018-0146-5 PMC5819194

[6] Sadath A Jose K Jiji KM : Prevalence and determinants of substance use among Indigenous tribes in South India: findings from a tribal household survey. *J Racial Ethn Health Disparities.* 2022;9(1):356–366. 33495925 10.1007/s40615-021-00964-2

[7] Browne AJ Varcoe C Lavoie J : Enhancing health care equity with Indigenous populations: evidence-based strategies from an ethnographic study. *BMC Health Serv Res.* 2016;16(1):544. 27716261 10.1186/s12913-016-1707-9 PMC5050637

[8] Prasad S Raushan R Tiwari R : Health status and healthcare care-seeking behavior: a study of tribal communities in Bijadandi block in Mandla district. *Int J Health Sci Res.* 2023;13(9):168–179. 10.52403/ijhsr.20230925

[9] Rowan M Poole N Shea B : Cultural interventions to treat addictions in Indigenous populations: findings from a scoping study. *Subst Abuse Treat Prev Policy.* 2014;9:34. 25179797 10.1186/1747-597X-9-34 PMC4158387

[10] Betancourt JR Green AR Carrillo JE : *Cultural competence in health care: emerging frameworks and practical approaches.* 2002; New York: Commonwealth Fund, Quality of Care for Underserved Populations:576. Reference Source

[11] Campinha-Bacote J : The process of cultural competence in the delivery of healthcare services: a model of care. *J Transcult Nurs.* 2002;13(3):181–184. 12113146 10.1177/10459602013003003

[12] Dale E Conigrave KM Kelly PJ : A Delphi yarn: applying Indigenous knowledges to enhance the cultural utility of SMART recovery Australia. *Addict Sci Clin Pract.* 2021;16(1):2. 33407873 10.1186/s13722-020-00212-8 PMC7787604

[13] Pandey M Clark M Beresh E : Wellness wheel clinics: a first nation community-partnered care model improving healthcare access, from healthcare providers’ perspectives. *Health Promot Int.* 2023;38(4):daad079. 37590386 10.1093/heapro/daad079

[14] Minkler M Wallerstein N : Community-based participatory research for health: from process to outcomes. 2nd ed. San Francisco: Jossey-Bass,2008. Reference Source

[15] World Health Organization: *International standards for the treatment of drug use disorders.* 2020; reviseded Geneva: WHO. Reference Source

[16] Rose A Minz S Manohari GP : Community perspectives on alcohol use among a tribal population in rural Southern India. *Natl Med J India.* 2015;28(3):117–121. 26724338

[17] Witkiewitz K Montes KS Schwebel FJ : What Is Recovery? *What is recovery? Alcohol Res.* 2020;40(3):arcr.v40.3.01. 32983748 10.35946/arcr.v40.3.01 PMC7505137

[18] Campbell C Jovchelovitch S : Health, community and development: towards a social psychology of participation. *J Community Appl Soc Psychol.* 2000;10(4):255–270. 10.1002/1099-1298(200007/08)10:4<255::AID-CASP582>3.0.CO;2-M

[19] Proctor E Silmere H Raghavan R : Outcomes for implementation research: conceptual distinctions, measurement challenges, and research agenda. *Adm Policy Ment Health.* 2011;38(2):65–76. 20957426 10.1007/s10488-010-0319-7 PMC3068522

[20] Guerrero EG Aarons GA Palinkas LA : Organisational capacity for service integration in community-based addiction health services. *Am J Public Health.* 2014;104(4):e40–e47. 24524525 10.2105/AJPH.2013.301842 PMC4020143

