# Supplementary material for: Formative evaluation of an existing community-based intervention for alcohol addiction in the Soliga tribal community in Chamarajanagar, Karnataka
Source: Wellcome Open Res. 2026 Oct 22;11:22. Originally published 2026 Jan 7. [Version 2] doi: 10.12688/wellcomeopenres.24075.2 (PMC13722858; doi:10.12688/wellcomeopenres.24075.2)
Supplement: Peer review history

# Reviewer report: Reviewer response for version 2

**DOI:** 10.21956/wellcomeopenres.30959.r175433

**Published:** 2026-10-30

**Authors:** Dr Srikanth Pallerla

**Affiliations:** 1 School of Sciences & Humanities, Sri Rajeshwara Educational Society, Warangal, Telangana, India

This version looks fine and can go ahead further

Is the study design appropriate for the research question?

Yes

Is the rationale for, and objectives of, the study clearly described?

Yes

Are sufficient details of the methods provided to allow replication by others?

Yes

Are the datasets clearly presented in a useable and accessible format?

Yes

Reviewer Expertise:

Rehabilitation, Psycho Social Interventions, Caregiver well being, Addiction management

I confirm that I have read this submission and believe that I have an appropriate level of expertise to confirm that it is of an acceptable scientific standard.

# Reviewer report: Reviewer response for version 2

**DOI:** 10.21956/wellcomeopenres.30959.r175432

**Published:** 2026-10-23

**Authors:** André J van Rensburg

**Affiliations:** 1 University of KwaZulu-Natal Howard Campus, Durban, South Africa

No further comments.

Is the study design appropriate for the research question?

Yes

Is the rationale for, and objectives of, the study clearly described?

Yes

Are sufficient details of the methods provided to allow replication by others?

Partly

Are the datasets clearly presented in a useable and accessible format?

Not applicable

Reviewer Expertise:

Public mental health; health system and policy research; integrated care; implementation science

I confirm that I have read this submission and believe that I have an appropriate level of expertise to confirm that it is of an acceptable scientific standard.

# Reviewer report: Reviewer response for version 1

**DOI:** 10.21956/wellcomeopenres.26561.r150947

**Published:** 2026-04-21

**Authors:** André J van Rensburg

**Affiliations:** 1 University of KwaZulu-Natal Howard Campus, Durban, South Africa

Thank you for the opportunity to review the submitted manuscript “Formative evaluation of an existing community-based intervention for alcohol addiction in the Soliga tribal community in Chamarajanagar, Karnataka”. The protocol speaks to a study of great importance, and has the potential to improve current understandings around responses to alcohol dependency in indigenous populations. The paper is succinct and clear, though there are some aspects which could be elaborated – suggestions are provided below:

- While this might go beyond the scope of a protocol, there is some space to provide more detail around the development of the intervention, in terms of approaches used, evidence drawn from, models applied, methods used etc.
- Given the history of mental health interventions being verticalized relative to broader systems of care, the authors are encouraged to reflect on how this intervention fits into broader systems, how well it is integrated with existing data flows, referral pathways etc.
- The mixed methods design is appropriate, but it is not clear how the phases are mixed, or when.
- Two key definitions are provided, namely for adoption and acceptability. This should be framed in terms of the well-established literature around these concepts – Proctor is cited, it might be useful reflecting on how these concepts are defined in implementation science, before adapting the definitions for local use.
- This intervention has multiple components, with multiple levels of implementation – what is the evidence for such an intervention as opposed to single component or level interventions?
- Would people with suicidal ideation be included?
- The last paragraph under Quantitative seems to miss crucial information, as it mentions that “The investigator will use free and open-source software (e.g. JAMOVI) to analyse the data gathered from various sources and measure acceptability and adoption using the following indicators”, without identifying the indicators.
- Given that recovery will be measured as well, it might be useful to use a validated instrument such as the RAS-DAS.

Is the study design appropriate for the research question?

Yes

Is the rationale for, and objectives of, the study clearly described?

Yes

Are sufficient details of the methods provided to allow replication by others?

Partly

Are the datasets clearly presented in a useable and accessible format?

Not applicable

Reviewer Expertise:

Public mental health; health system and policy research; integrated care; implementation science

I confirm that I have read this submission and believe that I have an appropriate level of expertise to confirm that it is of an acceptable scientific standard, however I have significant reservations, as outlined above.

# Response

**Published:** 2026-10-14

**Authors:** Alen Alexander

**Affiliations:** CITRITH, Institute of Public Health Bengaluru, Bengaluru, Karnataka, India

Thank you. Your suggestions have helped me build more context for the background of the intervention selected for the formative evaluation protocol and improve clarity in the Methodology section. I have listed your feedback below, along with the corresponding responses.

  

- ***While this might go beyond the scope of a protocol, there is some space to provide more detail around the developments of the intervention, in terms of approaches used, evidence drawn from, models applied, methods used etc. ***

Thank you for this suggestion. We have expanded the “History of Hosa Jeevana” section to provide further detail on how the intervention developed. We clarify that Hosa Jeevana did not originate from a pre-specified intervention-development framework, formal programme theory, or evidence-based model. Instead, it evolved organically through iterative service delivery in response to community members’ expressed needs and input, practitioners’ experiential knowledge, informal feedback, and observed barriers to care and engagement. We also explain that the programme theory was subsequently developed, as part of planning the formative evaluation, to articulate the intervention’s assumptions and anticipated mechanisms, and to guide its assessment and further refinement.

- ***Given the history of mental health interventions being verticalized relative to broader systems of care, the authors are encouraged to reflect on how this intervention fits into broader systems, how well it is integrated with existing data flows, referral pathways etc.***

Thank you for highlighting the importance of considering Hosa Jeevana (HJ) in relation to the broader system of care and the risk of delivering mental health and addiction interventions through vertical, programme-specific structures. We have added a subsection titled “Integration with existing systems of care” to the “Redesigned Hosa Jeevana” section. In this subsection, we clarify the relationship between HJ and the existing public-sector pathway. Although primary healthcare staff are expected to screen for and identify individuals requiring de-addiction care and refer them to the District Mental Health Programme (DMHP), the experiences informing HJ indicated that access through this pathway was constrained by inconsistent identification and referral at the primary-care level and by the geographical distance between Soliga settlements and available services. We therefore describe how HJ seeks to bridge this gap by linking community health workers, HJ champions, the tribal hospital, the clinical care coordinator, medical officers, and the DMHP psychiatrist. We have also provided further information on the intervention’s referral pathways and data flows. Screening information is transferred to the clinical care coordinator; enrolled individuals receive an HJ identification number and a hospital case file; and clinical and follow-up information is maintained in hospital and programme records. De-identified information is subsequently entered into a separate electronic database for the formative evaluation. We acknowledge that although these arrangements create functional links between community and clinical care, HJ’s referral and data flows are not yet fully embedded in routine public-sector referral and health information systems. The evaluation will examine how these links operate and identify opportunities to strengthen primary-care identification, bidirectional referral and feedback, shared documentation, and continuity of care.

- ***This intervention has multiple components, with multiple levels of implementation – what is the evidence for such an intervention as opposed to single component or level interventions?***

Thank you for raising this important question. We agree that the rationale for implementing the intervention across multiple components and levels required further explanation. Hosa Jeevana initially began primarily as a clinic-based intervention. The multicomponent design was not adopted from a single proven model or on the assumption that it was already known to be superior to a single-component intervention. Instead, the intervention expanded iteratively in response to limitations observed during the delivery of the initial clinic model and to input from community members, the community health worker, and other practitioners. These experiences identified barriers at several levels, including geographical access, community-level identification and referral, continuity of follow-up, culturally appropriate communication, and limited engagement with clinician-led support meetings. The redesigned intervention therefore includes clinical care, community screening and referral, peer support, follow-up, outreach, and culturally appropriate education, with each component intended to address a distinct identified barrier. We have revised the “Redesigned Hosa Jeevana” section to clarify this rationale. We also acknowledge that, although the individual components and underlying principles are supported by broader evidence, the combined HJ package has not been demonstrated to be superior to a single-component intervention in the Soliga context. Establishing comparative effectiveness is beyond the scope of this formative evaluation. The present study will instead examine the acceptability and adoption of the components, how they operate together in practice, and areas requiring refinement.

- ***The mixed methods design is appropriate, but it is not clear how the phases are mixed, or when. ***

Thank you for noting that the timing and integration of the two methodological strands required clarification. We have revised the manuscript to describe the study as an explanatory sequential mixed-methods design and to specify how and when mixing will occur. In the first phase, quantitative clinical and programme data will be analysed to describe patterns in referral acceptance, treatment initiation, engagement, retention, follow-up, and participation in community-level activities. The qualitative phase will commence approximately nine months after the quantitative phase begins, allowing sufficient programme data to accumulate for preliminary analysis. Preliminary quantitative findings will inform purposive selection of qualitative participants with varied experiences and levels of programme engagement and may be used to refine the emphasis of the interview guides. The qualitative interviews will then explore the barriers, facilitators, and contextual factors that may explain the quantitative patterns. We have also added a dedicated mixed methods integration section. Integration will occur by connecting preliminary quantitative findings to qualitative sampling, using quantitative patterns to refine the emphasis of qualitative inquiry, and comparing the separately analysed quantitative indicators and qualitative themes through an integration matrix. Areas of convergence, complementarity, and divergence will be examined to develop an integrated explanation of the intervention’s acceptability and adoption and identify areas requiring refinement.

- ***The last paragraph under Quantitative seems to miss crucial information, as it mentions that “The investigator will use free and open-source software (e.g. JAMOVI) to analyse the data gathered from various sources and measure acceptability and adoption using the following indicators”, without identifying the indicators.***

Thank you for identifying this lack of clarity. The acceptability and adoption indicators were presented later in Tables 2 and 3, but the final paragraph of the Quantitative Phase did not clearly direct readers to these tables or summarise the indicator domains. We have revised this paragraph to identify the principal quantitative indicators and explicitly cross-reference Tables 2 and 3. We have also clarified that quantitative indicators derived from clinical and programme records will be analysed using statistical software, whereas interview-derived information listed in the tables will be analysed thematically and integrated during the mixed-methods interpretation.

- ***Given that recovery will be measured as well, it might be useful to use a validated instrument such as the RAS-DAS.***

Thank you for this valuable suggestion. We agree that an instrument such as the RAS-DS could provide a broader assessment of personal, social, functional, and clinical recovery. In this formative evaluation, however, recovery is a secondary and exploratory outcome. It is assessed using alcohol-specific indicators, including changes in AUDIT scores, self-reported abstinence, relapse episodes, medication adherence, and intervals between relapses. Incorporating the RAS-DS would require assessing its relevance to alcohol recovery in this context, together with linguistic translation, cultural adaptation, and psychometric evaluation for use with the Soliga community. We have therefore not included it in the present study. We have added this limitation to the manuscript and will consider using an appropriately adapted multidimensional recovery instrument in a future study specifically designed to evaluate recovery outcomes.

- ***Would people with suicidal ideation be included? ***

Thank you for raising this important safety consideration. Suicidal ideation will not automatically exclude an individual from participation. We have added a safety procedure clarifying that interviewers will be trained to recognise participant distress. If a participant reports suicidal thoughts or other indications of immediate risk, the interview will be paused, and the clinical care coordinator or medical officer will be contacted for urgent assessment and referral. Confidentiality may be breached only to the extent required to protect the participant.

- ***Two key definitions are provided, namely for adoption and acceptability. This should be framed in terms of the well-established literature around these concepts – Proctor is cited, it might be useful reflecting on how these concepts are defined in implementation science, before adapting the definitions for local use. ***

Thank you for this important observation. We have revised the operational definitions to anchor acceptability and adoption explicitly in the implementation-science taxonomy described by Proctor et al. Acceptability is now defined as stakeholders’ perceptions that an intervention is agreeable, palatable, or satisfactory, while adoption is defined as the intention, initial decision, or action to try or employ an intervention. We then explain how these constructs are applied in the Hosa Jeevana context. Acceptability will be examined through stakeholder perceptions of satisfaction, comfort, usefulness, burden, and cultural and social responsiveness. Adoption will be examined through the uptake and use of HJ components by community members, HJ Champions, healthcare providers, and the participating healthcare institution. We have also clarified the relationship between these constructs and the selected indicators. Referral acceptance, treatment initiation, engagement, retention, and community participation will be treated as complementary behavioural indicators rather than direct measures of perceived acceptability. Indicators of routine integration and institutional embedding will be interpreted as related to penetration and potential sustainability rather than adoption alone. This revision preserves the locally relevant application while maintaining conceptual consistency with implementation-science literature.

# Reviewer report: Reviewer response for version 1

**DOI:** 10.21956/wellcomeopenres.26561.r150948

**Published:** 2026-03-26

**Authors:** Dr Srikanth Pallerla

**Affiliations:** 1 School of Sciences & Humanities, Sri Rajeshwara Educational Society, Warangal, Telangana, India

I appreciate the authors for choosing this research topic. The following observations are provided to strengthen the protocol, and necessary revisions may be made accordingly:

- The protocol needs to clearly specify the sample size for the qualitative component, as it includes multiple stakeholders such as patients, adult immediate family members, HJ Champions, community health workers, and clinical care coordinators. It should clarify whether a fixed number of participants (e.g., from each stakeholder group) will be selected or whether data collection will continue until saturation is reached within each group.
- Additionally, the protocol should explicitly indicate the number of stakeholders to be included in the qualitative phase.
- The exclusion criteria should not simply mirror the inclusion criteria; they need to be appropriately revised wherever required.
- Although inclusion and exclusion criteria for immediate family members are mentioned in the protocol, they should be clearly presented.
- For patients and family members, the inclusion criteria should specify age and language proficiency.
- The term “Immediate family members” should be replaced with “Adult immediate family members” for greater clarity.
- It should be specified whether the quantitative scales will be translated into the local tribal language for data collection.
- The protocol should mention that the developed interview guide will be shared with experts for content validation.
- The psychometric properties of the quantitative scales, including reliability and validity, should be reported.
- The strengths of the study should be clearly outlined.
- Information regarding funding should be included.
- Participant anonymity will be maintained by assigning unique identification codes and removing all personal identifiers.

Is the study design appropriate for the research question?

Yes

Is the rationale for, and objectives of, the study clearly described?

Yes

Are sufficient details of the methods provided to allow replication by others?

Yes

Are the datasets clearly presented in a useable and accessible format?

Yes

Reviewer Expertise:

Rehabilitation, Psycho Social Interventions, Caregiver well being, Addiction management

I confirm that I have read this submission and believe that I have an appropriate level of expertise to confirm that it is of an acceptable scientific standard, however I have significant reservations, as outlined above.

# Response

**Published:** 2026-10-14

**Authors:** Alen Alexander

**Affiliations:** CITRITH, Institute of Public Health Bengaluru, Bengaluru, Karnataka, India

Thank you. Your observations and suggested revisions to strengthen the protocol have certainly prompted me to revisit it and make some changes. I have listed your feedback below, along with the corresponding responses.

  

- ***It should be specified whether the quantitative scales will be translated into the local tribal language for data collection.*** Thank you for highlighting the need to specify the language used to administer the quantitative measure. The AUDIT is the only standardised quantitative scale used in this study; the remaining quantitative indicators are extracted from clinical and programme records. We have clarified that the HJ team translated and adapted the AUDIT into the colloquial Kannada routinely used by Soliga participants when communicating with health workers. Trained community health workers will administer the translated version orally when participants are unable to read or prefer oral administration. The same version will be used at baseline and follow-up to maintain consistency. We have not described this translated version as formally validated.
- ***The psychometric properties of the quantitative scales, including reliability and validity, should be reported.***
  Thank you for this suggestion. We have clarified that the AUDIT is internationally validated and has demonstrated acceptable psychometric properties in Indian populations. For this intervention, however, the AUDIT was translated and adapted into the colloquial Kannada that Soliga participants routinely use when communicating with health workers. The psychometric properties of this locally adapted version have not been independently established, and this limitation is now explicitly acknowledged in the manuscript. We have also clarified that the other quantitative indicators are derived from clinical and programme records rather than additional standardised scales.
- ***The protocol needs to clearly specify the sample size for the qualitative component, as it includes multiple stakeholders such as patients, adult immediate family members, HJ Champions, community health workers, and clinical care coordinators. It should clarify whether a fixed number of participants (e.g., from each stakeholder group) will be selected or whether data collection will continue until saturation is reached within each group.***
- ***Additionally, the protocol should explicitly indicate the number of stakeholders to be included in the qualitative phase. ***
- ***The exclusion criteria should not simply mirror the inclusion criteria; they need to be appropriately revised wherever required.***
- ***Although inclusion and exclusion criteria for immediate family members are mentioned in the protocol, they should be clearly presented. ***
- ***For patients and family members, the inclusion criteria should specify age and language proficiency. ***
- ***The term “Immediate family members” should be replaced with “Adult immediate family members” for greater clarity. ***
- ***The protocol should mention that the developed interview guide will be shared with experts for content validation.***
  Thank you for identifying the need to clarify the anticipated qualitative sample. We have revised the protocol to specify an anticipated sample of 12–15 current HJ participants, 8–10 adult immediate family members, 3–5 healthcare providers, 5–8 HJ Champions, and the clinical care coordinator, giving an estimated total of 29–39 stakeholders, subject to consent and availability. Healthcare providers may include community health workers and medical officers involved in HJ.
  Interviews with patients, adult immediate family members, and healthcare providers will continue within the anticipated ranges until thematic saturation is reached. Five to eight HJ Champions will be purposively selected based on their accessibility at the time of interview planning and to capture variation in their levels of programme participation. This variation may provide insights into the support and capacity-building required for Champions to perform their roles. The clinical care coordinator will be included as a key informant.
- ***Participant anonymity will be maintained by assigning unique identification codes and removing all personal identifiers.***
  Thank you for this suggestion. We have clarified the procedures for protecting participant confidentiality. Each participant will be assigned a unique study identification code, and direct personal identifiers will be removed from research records and datasets used for analysis. Any document linking participant identities to study identification codes will be stored separately and securely, with access restricted to authorised members of the study team. Data shared with collaborators or used in reports and publications will be de-identified. We have used the terms “confidentiality” and “de-identification” rather than “anonymity,” because authorised study-team members may retain access to the linkage information.
- ***The strengths of the study should be clearly outlined.***
  Thank you for this helpful comment. We have revised the Discussion section to include a paragraph explicitly outlining the study’s strengths. These include the evaluation of an existing community-based and culturally responsive intervention, the use of a mixed-methods design, the integration of routine program data with perspectives from multiple stakeholders, and the focus on implementation outcomes that can directly inform program refinement and service delivery.
- ***Information regarding funding should be included***
  Thank you for this comment. The funding information has been entered in the designated funding field on the journal’s submission platform and will be displayed alongside the manuscript on the published article webpage. We have verified that the information provided in this field is complete and accurate.
